# Conversion to mTOR-inhibitor-based immunosuppression: which patients and when?

**DOI:** 10.1186/2047-1440-2-S1-S3

**Published:** 2013-11-20

**Authors:** Philippe Gatault, Yvon Lebranchu

**Affiliations:** 1 CHRU Bretonneau, Service de Néphrologie et Immunologie Clinique, 2 Boulevard Tonnellé, 37000 Tours, France; 2 Université François-Rabelais, EA4245, 3 Rue des Tanneurs, 37000 Tours, France

**Keywords:** calcineurin inhibitors, conversion, immunosuppression, mammalian target of rapamycin inhibitors

## Abstract

Mammalian target of rapamycin (mTOR) inhibitors are currently considered an alternative immunosuppressive treatment that can prevent the nephrotoxicity, viral infections and malignancies that are associated with calcineurin inhibitor-based immunosuppressive regimens. However, the side effects of mTOR-inhibitor-based regimens lead to frequent treatment discontinuations, and not all patients seem to have the same benefits from conversion to mTOR inhibitors. This review focuses on long-term results of trials that have assessed early and late conversion to sirolimus or everolimus. The renal benefit of late conversion (≥1 year post transplantation) is limited, except in patients with good renal function and without proteinuria. Early conversion to mTOR inhibitors in the first 6 months, in combination with mycophenolate mofetil, could be an appropriate strategy for maintenance therapy in renal transplant recipients with a low immunological risk after careful screening at the time of conversion. Good renal function (glomerular filtration rate >40 ml/ minute), weak proteinuria (<1 g/day), an absence of previous acute rejection and subclinical rejection, and appearance of donor-specific anti-human leukocyte antigen antibodies appear to be the most important criteria in identifying patients for whom conversion to an mTOR inhibitor may improve renal function at 5 years.

## Introduction

Significant progress in organ transplantation in the past two decades has been mostly driven by improvement of short-term graft and patient survival due, in particular, to the use of calcineurin inhibitors (CNIs), which have reduced the rate of acute rejection considerably [1]. Nevertheless, this improvement in graft survival in the first year after transplantation has had a limited impact on long-term outcomes, which has only slowly improved [2]. This limited impact can be explained in part by the serious chronic adverse events associated with the use of CNIs, such as the increased risk of malignancies and cardiovascular events, which are the most frequent causes of death in kidney transplant patients. Importantly, CNIs also contribute to the development of chronic graft injuries [3]. Data suggest that CNI-sparing regimens could improve long-term graft and patient survival, as shown by Gallagher and colleagues, who reported improved 20-year graft survival in patients in whom cyclosporine (CsA) had been converted to azathioprine 3 months after transplantation in comparison with patients who continued CsA [4].

The advent of new immunosuppressive agents, such as mTOR inhibitors, has allowed CNI-based regimens to be used sparingly, and tests the hypothesis that CNIs contribute to chronic allograft nephropathy [5,6]. Sirolimus (SRL) binds to the mTOR complex and inhibits immune cell proliferation and differentiation. A pioneering trial of CNI withdrawal from SRL-based therapy demonstrated improved 4-year graft survival with improved renal function [7], showing that maintenance therapy with SRL and mycophenolate mofetil (MMF) was effective, thus paving the way to conversion strategies.

## Late conversion

In the CONVERT study, 830 patients were randomised 6 to 120 months after transplantation (mean 3.1 years) with a 2:1 ratio to either convert to SRL or to continue on a CNI (cyclosporine or tacrolimus) [8]. In addition, patients received steroids and adapted doses of either MMF or azathioprine. The primary endpoints were renal function, evaluated by the Nankivell glomerular filtration rate (GFR), and the cumulative rates of biopsy-proved acute rejection (BPAR), graft loss, or death at 12 months. Patients were stratified by baseline GFR: either 20 to 40 ml/minute or >40 ml/minute. Intent-to-treat analyses at 12 and 24 months showed no significant treatment differences in GFR. The mean GFR at 12 and 24 months was significantly higher in the group converted to SRL in comparison with the CNI group for patients with baseline GFR >40 ml/minute who remained on assigned therapy (63.6 vs. 61.1 ml/minute, *P* = 0.006 and 62.6 vs. 59.9 ml/minute, *P* = 0.009, at 12 and 24 months respectively) and for the subgroup with baseline GFR >40 ml/ minute and a urinary protein-to-creatinine ratio ≤0.11 (66.2 vs. 60.1 ml/minute, *P* = 0.004 and 63.8 vs. 59.0 ml/ minute, *P* = 0.049, at 12 and 24 months respectively). Graft and patient survival and the incidence of BPAR were similar in both groups. The discontinuation rate was higher in the SRL group at 12 months (15.7 vs. 9.5%, *P* = 0.013) but not at 24 months (25.8 vs. 20.0%, *P* = 0.07), with more adverse events during the first 6 months after randomisation. Interestingly, the incidence of malignancies was reduced after SRL conversion (3.8 vs. 11% at 24 months, *P* <0.001) [9].

A study of late conversion was performed with everolimus (EVL) [10]. In the ASCERTAIN study, 398 patients were randomised (mean 5.6 years after transplantation) to continue CNIs (cyclosporine or tacrolimus), to minimise CNI therapy with the addition of EVL or to convert to EVL. The mean measured GFR at 24 months, the primary endpoint, was not significantly different between the three groups, while proteinuria was significantly higher in the EVL group at 12 months. A *post-hoc* analysis in patients with better baseline graft function (defined by Nankivell GFR >50 ml/minute) and who remained on the randomised treatment regimen has shown that the increase in GFR from baseline to month 24 was significantly greater in the CNI elimination group than in control patients. Adverse events resulted in discontinuation for 28.3% of patients (*P* <0.001 vs. CNI-free patients) in the CNI elimination group, for 16.7% of patients in the CNI minimisation group (*P* = 0.02 vs. CNI-free patients) and for only 4% of patients who continued on a CNI-based regimen. The incidence of malignancies was not different between the three groups (7.1%, 7.6% and 5.7%, respectively).

These data suggest that the renal benefit of a late conversion, 1 year or more after transplantation, is limited, except in patients with good renal function and without proteinuria. Renal biopsy prior to conversion is useful to select patients without mild to severe chronic renal allograft damage in whom conversion from CNIs to mTOR inhibitors can be accomplished safely and effectively.

## Early conversion

Protocols of early CNI withdrawal with conversion to mTOR inhibitors in the maintenance phase have been performed with three main aims. The first is to achieve optimal renal function at 1 year, because long-term graft and patient survival have been associated with 1-year renal function [11-13]. A 10 ml/minute decrease in GFR at 1 year is associated with a 2.1 odds ratio of kidney allograft loss 3 years after transplantation [14].

The second aim is to reduce the incidence of viral infection, because previous studies have shown a low incidence of cytomegalovirus (CMV) infection in SRL-treated patients in comparison with CNI-treated patients [15]. A recent meta-analysis has shown that mTOR-inhibitor treatment, either alone or in combination with CNIs, significantly reduced the incidence of CMV infection after organ transplantation, suggesting that CMV prophylaxis may be dispensable with the use of mTOR inhibitors [16]. Furthermore, a significant increase in CMV-specific CD8^+^ T-cell count has been observed in EVL-treated renal recipients compared with CsA-treated patients [17], and functional mTOR has recently been reported to be essential to CMV replication, suggesting a direct antiviral effect of mTOR inhibitors [18]. A study has suggested that mTOR inhibitors also reduce the incidence of BK virus infection after transplantation [19].

The third aim is to decrease the incidence of malignancies. This aim is supported by several studies showing that mTOR-inhibitor-based regimens could reduce the incidence of neoplasia [20]. Moreover, it has recently been shown that conversion from a CNI to SRL in kidney transplant patients following a first skin cancer episode prevented the recurrence of skin cancer [21]. mTOR inhibitors have anti-neoplastic properties [22,23], in contrast to CNIs, which may induce cancer progression through mechanisms independent of host immunity [24].

Early conversion has been used in the CONCEPT study [25]. Two hundred and thirty-five nonimmunised patients transplanted with a deceased donor kidney received induction therapy with daclizumab and tri-therapy with CsA, MMF and steroids for 3 months. At 3 months, 192 patients with proteinuria <1 g/day and GFR ≥40 ml/ minute were randomised to either continue CsA (*n* = 97) or to convert to SRL (*n* = 95). MMF and steroids were planned to be discontinued at month 8.

Both groups were similar with respect to demographic and medical characteristics such as donor and recipient age, time of dialysis before transplantation, human leukocyte antigen and CMV matching, incidence of delayed graft function and GFR. The primary endpoint, estimated renal function (creatinine clearance) at 1 year according to the Cockcroft–Gault equation, was significantly better in the SRL group (68.9 vs. 64.4 ml/minute, *P* = 0.017). Similar results were observed when the GFR was calculated according to the Modification of Diet in Renal Disease formula (61.2 vs. 53.9 ml/minute, *P* = 0.002) or was measured using iohexol (67.3 vs. 60.3 ml/minute, *P* = 0.004). Patient and graft survival were excellent, with no death and only one graft loss, which occurred in the CsA group. CsA and SRL dosages and levels were adapted at 12 months to a mean daily dosage of 226 mg CsA, with mean blood levels 2 hours after dosing of 749 ng/ml, and to a mean daily dosage of 3.2 mg SRL with a mean trough level of 9.6 ng/ml.

The incidence of BPAR episodes was not significantly higher in the SRL group (17% vs. 8%, *P* = 0.07), while steroids were withdrawn in 72% and 78% of patients, respectively. Of note, most episodes of BPAR occurred just after withdrawal of steroids in the SRL group. The incidence of adverse events (stomatitis, acne, diarrhoea, high triglyceride levels) was slightly increased in the SRL group (60% vs. 44%, *P* = 0.025) and more patients discontinued SRL (16% vs. 7%). Interestingly, haemoglobin, cholesterol, and proteinuria were similar in both groups. The number of patients with proteinuria >0.5 g/ day was also similar in both groups (12% in the SRL group vs. 9% in the CsA group). Some adverse events required adjustment of the MMF daily dose (1.7 g/day in the SRL group vs. 1.9 g/day in the CsA group, *P* <0.001). Aortic stiffness and biomarkers of endothelial activation were studied in 44 patients enrolled in the CONCEPT study [26]. One year after transplantation, the carotid-to-femoral pulse-wave velocity was significantly lower in the SRL group. In parallel, plasma levels of endothelin-1 decreased in the SRL group during the study, suggesting a beneficial effect of SRL in preventing the development of cardiovascular complications after kidney transplantation. Conversion from CsA to SRL combined with MMF treatment 3 months after transplantation was therefore associated with an improvement in renal function with a good risk-to-benefit ratio.

Other studies have confirmed the CONCEPT study results, irrespective of the mTOR inhibitor used. At 1 year, the renal benefit of early conversion from CNIs to mTOR inhibitors has been observed with both SRL [27,28] and EVL [29]. In the Spare-the-Nephron study, 299 patients were randomised 1 to 6 months after transplantation (mean 3.8 months) to continue CNI therapy or to convert to SRL (CsA, *n* = 31 or tacrolimus, *n* = 120) [28]. After 1 year, the mean percentage change from baseline of measured GFR was significantly higher in the MMF/SRL group compared with the MMF/CNI group (24.4% vs. 5.2%, *P* = 0.012). The GFR, calculated according to Nankivell, was higher in the SRL group but the difference was not significant (74.6 vs. 71.5 ml/ minute). In the SMART study, 161 patients with a low to moderate immunological risk were randomised 10 to 24 days after transplantation to convert to SRL or to continue CsA [27]. The primary endpoint, renal function estimated at 1 year according to Nankivell, was significantly better in the SRL group (64.5 vs. 53.4 ml/minute, *P* = 0.0019). In the ZEUS trial, 300 patients were randomised at 4.5 months to continue CsA or be converted to EVL [29]. At 1 year, the EVL regimen was associated with a better renal function evaluated according to Nankivell (71.8 vs. 61.9 ml/minute, *P* <0.0001). Similar results were reported in the HERAKLES study at the last meeting of the American Congress of Transplantation [30]. The percentage of BPAR at 1 year was low and similar in both groups in these studies (11.3% vs. 9.5% in Spare-the-Nephron, 17% vs. 16% in SMART, 15% vs. 15% in ZEUS). Nevertheless, a significantly increased incidence of BPAR was reported in the EVL group in the randomised period in the ZEUS trial (10% vs. 6%, *P* = 0.04). One-year graft and patient survival were similar in both groups in all studies. However, more adverse events and more discontinuations were observed after conversion to mTOR inhibitors. These studies (SMART, ZEUS, HERAKLES) assessing substitution of CsA with an mTOR inhibitor show that the renal benefit at 1 year (about 8 to 10 ml/ minute) was similar to those observed in CONCEPT, whereas it was reduced with tacrolimus [28,31].

Heilman and colleagues have reported a prospective, randomised, nonblinded trial of early tacrolimus elimination at 1 month (60 and 62 patients in the tacrolimus and SRL groups, respectively) [31]. In this study, the measured GFR was similar in both groups at 1 and 2 years. Incidence of acute rejection was higher in the SRL group than in the tacrolimus group (Banff ≥IA: 13% vs. 5%, *P* = 0.15). Nevertheless, a very high percentage of withdrawal was observed in the SRL group (63% during the 2-year period). Other studies comparing the efficacy and safety of mTOR inhibitors with tacrolimus do not support the advantages of mTORs [32-34]. Nevertheless, in all of these studies, mTOR inhibitors were used at transplantation (*de novo*) with a high percentage of early withdrawal due to adverse events and sometimes subtherapeutic dosing, especially in the Symphony study.

Early conversion to SRL with continuation of MMF may therefore represent an appropriate strategy for maintenance therapy in renal transplantation after careful screening at the time of transplantation. From these studies we can consider that the more suitable patients for early conversion are nonimmunised patients with good renal function (GFR >40 ml/minute), without previous severe acute rejection and subclinical rejection, in the absence of proteinuria >1 g/day and with donor-specific antibodies. Screening biopsy prior to conversion is important in selecting appropriate patients.

## Long-term clinical outcomes

Long-term clinical outcome studies are necessary to confirm the short-term benefits of early CNI withdrawal. Patients who completed the initial 12 months of the SPIESSER and the CONCEPT studies were therefore enrolled in the post-SPIESSER and post-CONCEPT follow-up studies [35,36].

**Figure 1:**
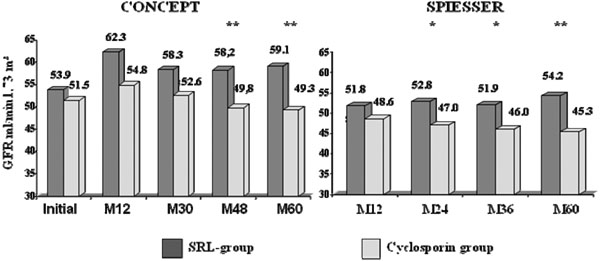
**Intent-to-treat analysis of the estimated glomerular filtration rate.** Analysis according to the Modification of Diet in Renal Disease formula in the CONCEPT study (left) and in the SPIESSER study (right). *P <0.05, **P <0.01. GFR, glomerular filtration rate; M, month; SRL, sirolimus.

**Figure 2:**
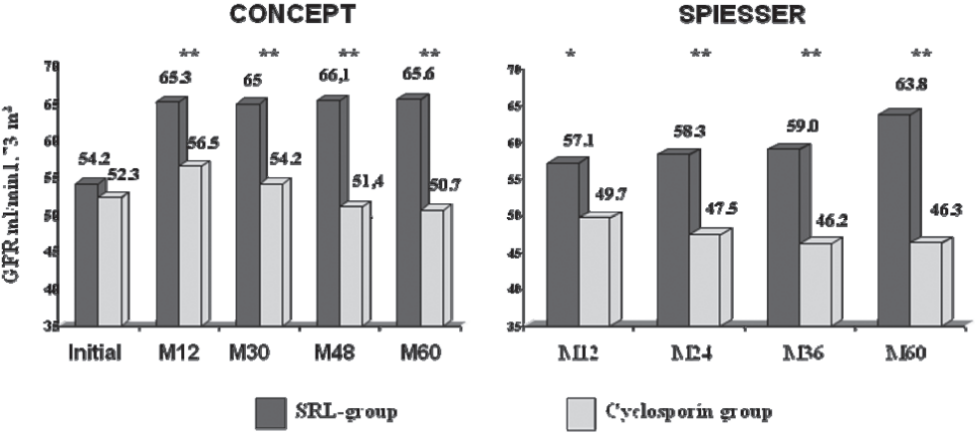
**On-treatment analysis of the estimated glomerular filtration rate.** Analysis according to the Modification of Diet in Renal Disease formula in the CONCEPT study (left) and the SPIESSER study (right), **P <0.01. GFR, glomerular filtration rate; M, month; SRL, sirolimus.

The 5-year results have been evaluated in 135 patients in the post-CONCEPT study (SRL, *n* = 65 and CsA, *n* = 70) and 130 patients in the post-SPIESSER study (SRL, *n* = 57 and CsA, *n* = 63). Patient survival and death-censored graft survival were excellent in both studies and similar in both groups. In the SRL groups in the post-SPIESSER and post-CONCEPT studies, patient survival was 93% and 97.4% and death-censored graft survival was 87% and 97.4%. However, the benefit on renal function in the SRL group, observed at 1 year, was maintained over 5 years in both studies (Figure 1). Renal function was significantly better in the SRL group in both studies in the intent-to-treat populations. The 5-year mean GFR, estimated according to the Modification of Diet in Renal Disease formula, was 59.1 versus 49.3 ml/ minute (*P* = 0.0012) in the post-CONCEPT study and 54.5 versus 45.3 ml/minute (*P* <0.01) in the post-SPIESSER study. Interestingly, this difference was more pronounced in patients who remained in their randomised arm at year 5 (Figure 2), with a 14.9 ml/minute and a 17.5 ml/minute difference in the CONCEPT and SPIESSER studies, respectively. Moreover, a negative GFR slope with a progressive deterioration of renal function was observed in patients who received CsA in both studies, but was not seen in the SRL groups.

Mean daily SRL and CsA doses and trough levels of SRL were similar in the SPIESSER and CONCEPT studies (SRL doses, 2.7 and 2.4 mg/day; SRL levels, 8.7 and 7.6 ng/ml; and CsA doses, 177 and 170 mg/day in the two studies respectively). In both studies, daily doses of MMF were adapted (CsA groups, 1,587 and 1,825 mg/ day; SRL groups, 1,403 and 1,542 mg/day in the two studies respectively). Interestingly, the percentage of steroid-free patients was higher in the SRL groups (76% and 73% vs. 69% and 61% in the two studies respectively). The occurrence of BPAR after 1 year was low in both studies (2 and 2 vs. 2 and 6 in the SRL and CsA groups from the SPIESSER and CONCEPT studies, respectively). The rate of patients with anti-human leukocyte antigen at 5 years was also similar (22% and 12.3% vs. 16% and 21.1% respectively). The 15% increased incidence of discontinuations observed at 1 year in the SRL groups was maintained at 5 years (40% and 44.6% vs. 24.2% and 21.6% respectively), with an increased incidence of side effects such as oedema, stomatitis, pneumonia and pyelonephritis. More patients with new-onset diabetes after transplantation were observed in the SRL group in the CONCEPT study but not in the SPIESSER study. The number of patients who developed malignancies (that is, skin cancers and nonskin malignancies) during follow-up was higher in the CsA groups in both studies in the ITT populations (9 and 9 vs. 4 and 6 respectively).

Lipid values (total cholesterol, low-density lipoprotein-cholesterol, high-density lipoprotein-cholesterol and triglycerides) and the percentage of patients receiving lipid-lowering agents were similar at 5 years in the two treatment groups in both studies.

There were no differences in haemoglobin values, either in the percentage of anaemic patients (defined as haemoglobin value <11 g/dl) or in the percentage of patients receiving an erythropoietin-stimulating agent between groups in both studies. However, mean red blood cell counts were higher in the SRL group, whereas mean corpuscular volumes were lower. Interestingly, mean proteinuria was similar in both groups at 5 years in both studies (0.5 and 0.4 g/24 hours vs. 0.4 and 0.4 g/24 hours). Moreover, the percentage of patients with proteinuria >0.3 g/24 hours and the percentage of patients treated either with an angiotensin-converting-enzyme inhibitor and/or an angiotensin-receptor blocker were similar in both groups.

The 5-year results for CNI elimination with a SRL and MMF regimen therefore demonstrated that the renal benefit observed 1 year after transplantation was maintained and even increased with stability in the GFR in patients remaining on assigned SRL therapy – compared with patients remaining on assigned CsA therapy, in whom the GFR was progressively declining. Moreover, fewer malignancies were observed. These benefits were observed despite more SRL discontinuations due to early adverse events. Of note, similar long-term benefit was observed with the CNI-free regimen [37].

## Conclusion

Early conversion to mTOR inhibitors in combination with MMF could be an appropriate strategy for maintenance therapy in renal transplant recipients with a low immunological risk, after careful screening at the time of conversion. Whether the benefits observed in these trials could influence long-term graft and patient survival remain to be determined.

## Abbreviations

BPAR: biopsy-proved acute rejection; CMV: cytomegalovirus; CNI: calcineurin nhibitor; CsA: cyclosporine; EVL: everolimus; GFR: glomerular filtration rate; MMF: mycophenolate mofetil; mTOR: mammalian target of rapamycin; SRL: sirolimus.

## Competing interests

YL has received grants, consultancy and lecture fees from Alexion, Astellas, LFB, Novartis, Pfizer, Roche BMS, and Sanofi. PG declares that he has no competing interests.

## Declaration

This article has been published as part of *Transplantation Research* Volume 2 Suppl 1, 2013: Proceedings of the 12th International Symposium on Transplantation. The full contents of the supplement are available at http://www.transplantationresearch.com/supplements/2/S1. The supplement is based on the 12th International Transplantation Symposium held on 16–17 April 2012, in Istanbul, Turkey. The symposium and publication based on these proceedings were supported by an unrestricted educational grant from Pfizer Inc., who had no influence on the editorial content. Editorial support was provided by Synergy, Richmond, UK and was funded by Pfizer Inc. Rapamune (sirolimus) is a product marketed by Pfizer Inc. The articles have been through the journal’s standard peer review process and the Supplement Editors declare that they have no competing interests.

## References

[B1] Meier-Kriesche HU Schold JD Srinivas TR Kaplan B Lack of improvement in renal allograft survival despite a marked decrease in acute rejection rates over the most recent era Am J Transplant 2004 2 378 383 10.1111/j.1600-6143.2004.00332.x 14961990

[B2] Lamb KE Lodhi S Meier-Kriesche HU Long-term renal allograft survival in the United States: a critical reappraisal Am J Transplant 2011 2 450 462 10.1111/j.1600-6143.2010.03283.x 20973913

[B3] Nankivell BJ Borrows RJ Fung CL O’Connell PJ Allen RD Chapman JR The natural history of chronic allograft nephropathy N Engl J Med 2003 2 2326 2333 10.1056/NEJMoa020009 14668458

[B4] Gallagher M Jardine M Perkovic V Cass A McDonald S Petrie J Eris J Cyclosporine withdrawal improves long-term graft survival in renal transplantation Transplantation 2009 2 1877 1883 10.1097/TP.0b013e3181a76823 19543068

[B5] Chapman JR O’Connell PJ Nankivell BJ Chronic renal allograft dysfunction J Am Soc Nephrol 2005 2 3015 3026 10.1681/ASN.2005050463 16120819

[B6] Guerra G Srinivas TR Meier-Kriesche HU Calcineurin inhibitor-free immunosuppression in kidney transplantation Transpl lnt 2007 2 813 827 10.1111/j.1432-2277.2007.00528.x 17645419

[B7] Oberbauer R Segoloni G Campistol JM Kreis H Mota A Lawen J Russ G Grinyó JM Stallone G Hartmann A Pinto JR Chapman J Burke JT Brault Y Neylan JF Rapamune Maintenance Regimen Study Group Early cyclosporine withdrawal from a sirolimus-based regimen results in better renal allograft survival and renal function at 48 months after transplantation Transpl lnt 2005 2 22 28 10.1111/j.1432-2277.2004.00052.x 15612979

[B8] Schena FP Pascoe MD Alberu J del Carmen Rial M Oberbauer R Brennan DC Campistol JM Racusen L Polinsky MS Goldberg-Alberts R Li H Scarola J Neylan JF Sirolimus CONVERT Trial Study Group Conversion from calcineurin inhibitors to sirolimus maintenance therapy in renal allograft recipients: 24-month efficacy and safety results from the CONVERT trial Transplantation 2009 2 233 224 10.1097/TP.0b013e3181927a41 19155978

[B9] Alberu J Pascoe MD Campistol JM Schena FP Rial Mdel C Polinsky M Neylan JF Korth-Bradley J Goldberg-Alberts R Mailer ES Sirolimus CONVERT Trial Study Group Lower malignancy rates in renal allograft recipients converted to sirolimus-based, calcineurin inhibitor-free immunotherapy: 24-month results from the CONVERT trial Transplantation 2011 2 303 310 10.1097/TP.0b013e3182247ae2 21792049

[B10] Holdaas H Rostaing L Seron D Cole E Chapman J Fellstrom B Strom EH Jardine A Midtvedt K Machein U Ulbricht B Karpov A O’Connell PJ ASCERTAIN Investigators Conversion of long-term kidney transplant recipients from calcineurin inhibitor therapy to everolimus: a randomized, multicenter, 24-month study Transplantation 2011 2 410 418 10.1097/TP.0b013e318224c12d 21697773

[B11] Go AS Chertow GM Fan D McCulloch CE Hsu CY Chronic kidney disease and the risks of death, cardiovascular events, and hospitalization N Engl J Med 2004 2 1296 1305 10.1056/NEJMoa041031 15385656

[B12] Marcen R Pascual J Tenorio M Ocana EJ Teruel JL Villafruela JJ Fernández M Burgos FJ Ortuño J Chronic kidney disease in renal transplant recipients Transplant Proc 2005 2 3718 3720 10.1016/j.transproceed.2005.09.101 16386516

[B13] Hariharan S Kasiske B Matas A Cohen A Harmon W Rabb H Surrogate markers for long-term renal allograft survival Am J Transplant 2004 2 1179 1183 10.1111/j.1600-6143.2004.00484.x 15196079

[B14] Ekberg H Bernasconi C Tedesco-Silva H Vitko S Hugo C Demirbas A Acevedo RR Grinyó J Frei U Vanrenterghem Y Daloze P Halloran P Calcineurin inhibitor minimization in the Symphony study: observational results 3 years after transplantation Am J Transplant 2009 2 1876 1885 10.1111/j.1600-6143.2009.02726.x 19563339

[B15] Buchler M Caillard S Barbier S Thervet E Toupance O Mazouz H Hurault de Ligny B Le Meur Y Thierry A Villemain F Heng AE Moulin B Morin MP Noël C Lebranchu Y SPIESSER Group Sirolimus versus cyclosporine in kidney recipients receiving thymoglobulin, mycophenolate mofetil and a 6-month course of steroids Am J Transplant 2007 2 2522 2531 10.1111/j.1600-6143.2007.01976.x 17868057

[B16] Andrassy J Hoffmann VS Rentsch M Stangl M Habicht A Meiser B Fischereder M Jauch KW Guba M Is cytomegalovirus prophylaxis dispensable in patients receiving an mTOR inhibitor-based immunosuppression? A systematic review and meta-analysis Transplantation 2012 2 1208 1217 23269449 10.1097/TP.0b013e3182708e56

[B17] Havenith SH Yong SL van Donselaar-van der Pant KA van Lier RA Ten Berge IJ Bemelman FJ Everolimus-treated renal transplant recipients have a more robust CMV-specific CD8^+^T-cell response compared with cyclosporine- or mycophenolate-treated patients Transplantation 2013 2 184 191 10.1097/TP.0b013e318276a1ef 23222818

[B18] Poglitsch M Weichhart T Hecking M Werzowa J Katholnig K Antlanger M Krmpotic A Jonjic S Hörl WH Zlabinger GJ Puchhammer E Säemann MD CMV late phase-induced mTOR activation is essential for efficient virus replication in polarized human macrophages Am J Transplant 2012 2 1458 1468 10.1111/j.1600-6143.2012.04002.x 22390651

[B19] Benavides CA Pollard VB Mauiyyedi S Podder H Knight R Kahan BD BK virus-associated nephropathy in sirolimus-treated renal transplant patients: incidence, course, and clinical outcomes Transplantation 2007 2 83 88 10.1097/01.tp.0000268524.27506.39 17627242

[B20] Kreis H Oberbauer R Campistol JM Mathew T Daloze P Schena FP Burke JT Brault Y Gioud-Paquet M Scarola JA Neylan JF Rapamune Maintenance Regimen Trial Long-term benefits with sirolimus-based therapy after early cyclosporine withdrawal J Am Soc Nephrol 2004 2 809 817 10.1097/01.ASN.0000113248.59077.76 14978184

[B21] Euvrard S Morelon E Rostaing L Goffin E Brocard A Tromme I Broeders N del Marmol V Chatelet V Dompmartin A Kessler M Serra AL Hofbauer GF Pouteil-Noble C Campistol JM Kanitakis J Roux AS Decullier E Dantal J TUMORAPA Study Group Sirolimus and secondary skin-cancer prevention in kidney transplantation N Engl J Med 2012 2 329 339 10.1056/NEJMoa1204166 22830463

[B22] Geissler EK Schlitt HJ Thomas G mTOR, cancer and transplantation Am J Transplant 2008 2 2212 2218 10.1111/j.1600-6143.2008.02391.x 18785960

[B23] Koehl GE Andrassy J Guba M Richter S Kroemer A Scherer MN Steinbauer M Graeb C Schlitt HJ Jauch KW Geissler EK Rapamycin protects allografts from rejection while simultaneously attacking tumors in immunosuppressed mice Transplantation 2004 2 1319 1326 10.1097/00007890-200405150-00002 15167584

[B24] Hojo M Morimoto T Maluccio M Asano T Morimoto K Lagman M Shimbo T Suthanthiran M Cyclosporine induces cancer progression by a cell-autonomous mechanism Nature 1999 2 530 534 10.1038/17401 10028970

[B25] Lebranchu Y Thierry A Toupance O Westeel PF Etienne l Thervet E Moulin B Frouget T Le Meur Y Glotz D Heng AE Onno C Buchler M Girardot-Seguin S Hurault de Ligny B Efficacy on renal function of early conversion from cyclosporine to sirolimus 3 months after renal transplantation: concept study Am J Transplant 2009 2 1115 1123 10.1111/j.1600-6143.2009.02615.x 19422337

[B26] Joannides R Monteil C de Ligny BH Westeel PF lacob M Thervet E Barbier S Bellien J Lebranchu Y Seguin SG Thuillez C Godin M Etienne I Immunosuppressant regimen based on sirolimus decreases aortic stiffness in renal transplant recipients in comparison to cyclosporine Am J Transplant 2011 2 2414 2422 10.1111/j.1600-6143.2011.03697.x 21929645

[B27] Guba M Pratschke J Hugo C Kramer BK Nohr-Westphal C Brockmann J Andrassy J Reinke P Pressmar K Hakenberg O Fischereder M Pascher A Illner WD Banas B Jauch KW SMART-Study Group Renal function, efficacy, and safety of sirolimus and mycophenolate mofetil after short-term calcineurin inhibitor-based quadruple therapy in de novo renal transplant patients: one-year analysis of a randomized multicenter trial Transplantation 2010 2 175 183 20463641 10.1097/TP.0b013e3181e11798

[B28] Weir MR Mulgaonkar S Chan L Shidban H Waid TH Preston D Kalil RN Pearson TC Mycophenolate mofetil-based immunosuppression with sirolimus in renal transplantation: a randomized, controlled Spare-the-Nephron trial Kidney Int 2011 2 897 907 10.1038/ki.2010.492 21191361

[B29] Budde K Becker T Arns W Sommerer C Reinke P Eisenberger U Kramer S Fischer W Gschaidmeier H Pietruck F ZEUS Study Investigators Everolimus-based, calcineurin-inhibitor-free regimen in recipients of de-novo kidney transplants: an open-label, randomised, controlled trial Lancet 2011 2 837 847 10.1016/S0140-6736(10)62318-5 21334736

[B30] Budde K Witzke O Lehner F Zeier M Neumayer HN Stangl M Jacobi J Kliem V May C Paulus EM Arns W Sommerer C Superior renal function in an everolimus-based calcineurin inhibitor free regimen compared to standard cyclosporine/mycophenolate and low cyclosporine/everolimus: the HERAKLES Study [abstract #50] Am J Transplant 2012 2 Suppl3 abstract #5022553852

[B31] Heilman RL Younan K Wadei HM Mai ML Reddy KS Chakkera HA Gonwa TA Results of a prospective randomized trial of sirolimus conversion in kidney transplant recipients on early corticosteroid withdrawal Transplantation 2011 2 767 773 10.1097/TP.0b013e31822805d7 21775930

[B32] Larson TS Dean PG Stegall MD Griffin MD Textor SC Schwab TR Gloor JM Cosio FG Lund WJ Kremers WK Nyberg SL Ishitani MB Prieto M Velosa JA Complete avoidance of calcineurin inhibitors in renal transplantation: a randomized trial comparing sirolimus and tacrolimus Am J Transplant 2006 2 514 522 10.1111/j.1600-6143.2005.01177.x 16468960

[B33] Ekberg H Tedesco-Silva H Demirbas A Vitko S Nashan B Gürkan A Margreiter R Hugo C Grinyó JM Frei U Vanrenterghem Y Daloze P Halloran PF ELITE-Symphony Study: reduced exposure to calcineurin inhibitors in renal transplantation N Engl J Med 2007 2 2562 2575 10.1056/NEJMoa067411 18094377

[B34] Flechner SM Glyda M Cockfield S Grinyo J Legendre CH Russ G Steinberg S Wissing KM Tai SS The ORION study: comparison of two sirolimus-based regimens versus tacrolimus and mycophenolate mofetil in renal allograft recipients Am J Transplant 2011 2 1633 1644 10.1111/j.1600-6143.2011.03573.x 21668635

[B35] Lebranchu Y Thierry A Thervet E Buchler M Etienne I Westeel PF Hurault de Ligny B Moulin B Rérolle JP Frouget T Girardot-Seguin S Toupance O Efficacy and safety of early cyclosporine conversion to sirolimus with continued MMF-four-year results of the Postconcept study Am J Transplant 2011 2 1665 1675 10.1111/j.1600-6143.2011.03637.x 21797975

[B36] Lebranchu Y Snanoudj R Toupance O Weestel PF Hurault de Ligny B Buchler M Rerolle JP Thierry A Moulin B Subra JF Deteix P Le Pogamp P Finzi L Etienne I Five-year results of a randomized trial comparing de novo sirolimus and cyclosporine in renal transplantation: the SPIESSER study Am J Transplant 2012 2 1801 1810 10.1111/j.1600-6143.2012.04036.x 22486815

[B37] Flechner SM Goldfarb D Solez K Modlin CS Mastroianni B Savas K Babineau D Kurian S Salomon D Novick AC Cook DJ Kidney tranplantation with sirolimus and mycophenolate mofetil-based immunosuppression: 5-year results of a randomized prospective trial compared to calcineurin inhibitor drugs Transplantation 2007 2 883 892 10.1097/01.tp.0000258586.52777.4c 17460558

